# Towards H&E Referenced Multiplex Immunofluorescence Interpretation: Spatial Co-localization, Cell Feature Validation, and Virtual H&E Generation

**DOI:** 10.21203/rs.3.rs-5619126/v1

**Published:** 2025-01-06

**Authors:** Chen Wang, Jun Jiang, Raymond Moore, Brenna Novotny, Ruifeng Guo, Zachary Fogarty, Yuanhang Liu, Ellen Goode, Stacey Winham, Svetomir Markovic

**Affiliations:** Mayo Clinic; Mayo Clinic; Mayo Clinic; Mayo Clinic; Mayo Clinic; Mayo Clinic; Mayo Clinic; Mayo Clinic; Mayo Clinic; Mayo Clinic

**Keywords:** Histopathology alignment, Histopathology registration, Bioimage analysis

## Abstract

Multiplexed Immunofluorescence (MxIF) enables detailed immune cell phenotyping, providing critical insights into cell behavior within the tumor immune microenvironment (TIME). However, signal integrity can be compromised due to the complex cyclic staining processes inherent to MxIF. Hematoxylin and Eosin (H&E) staining, on the other hand, offers complementary information through its depiction of cell morphology and texture patterns and is often visually cross-referenced with MxIF in clinical settings. In this study, we proposed a novel framework to align H&E and MxIF images for precise cross-modal cell feature validation. Using cell detection outputs from each modality as anchors, we formulated the multimodal image registration problem as point set alignment. Coherent Point Drift (CPD) is employed for initial alignment, followed by Graph Matching (GM) for refinement. Evaluations on ovarian cancer tissue microarrays (TMAs) demonstrate that our method achieves high alignment accuracy, enabling reliable validation of cell-level features across modalities for both restained and serial sections. Our results indicate that restained H&E enhances confidence in findings derived from MxIF. Additionally, we demonstrated the feasibility of generating high-quality virtual H&E images from MxIF data when restained H&E is unavailable, offering a viable alternative for integrated multimodal analysis.

## Introduction

As an important approach to reveal cell level details in cancer, histopathological images have been widely used in both clinic practice for diagnostic decision making and treatment follow up. Following different staining protocols, each modality of histopathology has its unique strength in highlighting specific aspects within the tumor immune microenvironment (TIME). Among them, multiplexed Immunofluorescence (MxIF) images provide refined immune cell phenotyping, making it a favorable research tool for revealing cell behaviors in TIME. However, MxIF has yet to see widespread adoption in clinical practice, largely due to challenges in maintaining marker signal integrity during the complex cyclic staining processes. On the other hand, H&E (Hematoxylin and Eosin) staining plays an irreplaceable role in providing standard clinical references by revealing cell morphology and texture patterns. Thus, accumulating studies have been combining cell level information from H&E with other histopathology stains to interrogate the mechanism of cancer development and metastasis. For example, Bao etc. proposed a bifocal neural network to explicitly learn from H&E and IHC (Immunohistochemistry) for identifying abnormalities in pathology images [1]. Gatenbee etc. [2, 3] compared the characterization of TIME using MxIF and H&E. Overall, the prerequisite of integrative multimodal histopathology TIME analysis is to establish cell scale correspondence between multimodalities, as the combined cell level characterization relies on co-localized cells within cancer tissues.

Although it is feasible to align diverse histopathological staining by annotating a few landmarks, this labor-intensive labeling work makes it hard to be applied to all the images in a large cohort study, especially for tissue micro-arrays (TMAs), as each whole slide image (WSI) contains hundreds of tissue cores. Image registration is an automatic technique that has been widely used for medical image alignment to establish pixel level correspondence. For example. Jonsson etc. [4] developed an image registration method targeted towards computer-aided voxel-wise analysis of whole-body PET-CT data. Jiang etc. developed an automatic alignment method by capturing the hierarchical nature of whole slide images [5]. Although image registration has been leveraged in histopathology images, it is still challenging to achieve cell-level alignment for the following reasons. First, the size of histopathology images (usually more than 3GB) is much larger than other medical images, such as CT or MR (usually less than 1MB), which means many existing methods can’t be directly applied in our scenario due to the limitation of computational resource. Second, the contents (cells) within histopathology imaging are dense and small compared to the image size, which exhausts the algorithm to converge; Third, the content of images acquired from diverse modalities can be dramatically different. Some cells in one modality may not match the compartments in another modality even if the images are from the restained tissue section, as the pigment only bonds to cells with specific markers. In consequence, algorithms may not be able to find enough reliable landmarks to establish the location correspondence. Thus, there exists an urgent need for multimodal histopathology alignment to enable integrating detailed information from different stains.

Given the challenges of image alignment by directly using pixel information, it is straightforward to consider using cell segmentation outcomes from histopathology images as the starting point to develop approaches for establishing cell spatial correspondence. This resolving path does not incur extra cost to the existing workflow since cell segmentation is the premise of cell level downstream spatial analysis. On the contrary, it can significantly reduce computational complexity as the number of cells is dramatically less than the number of pixels within a histopathology image. Along this resolving path, two major components are required: 1) cell segmentation; 2) point set alignment. Cell segmentation denotes the methods to detect cells and locate the boundaries of cells from histopathological images. With the advancement of AI and Deep Learning, many cell segmentation models achieve not only superior performance but also high generalizability and robustness[6–8]. Since cell segmentation is comparable to the key points detection steps within the traditional image registration process, the advancement in AI-enhanced cell segmentation can be fully leveraged in this solving path. With the centroids of cell segmentations, cells within histopathology images can be summarized as a point set. Thus, establishing cell spatial correspondence can be formulated as a point sets alignment problem, which has also been widely explored within the machine learning and pattern recognition field [9, 10].

In this paper, we present a novel framework to establish spatial correspondence for cells from different histopathology stainings, allowing us to validate cell features across different modalities and enabling the synergy of combinational analysis. By formulating the cell spatial correspondence into point sets alignment, we used cell segmentation results from histopathology image pairs as the basis, cells were first aligned with Coherent Point Drift (CPD) [10] and then the alignment was calibrated using Graph Matching (GM)[11]. We systematically evaluated the alignment accuracy for both restained and serial section images from ovarian cancer TMA images. The proposed method achieves encouraging alignment accuracy and is portable to different cell segmentation methods. Within the aligned multimodal image pairs, we found that cell morphology and nucleus staining features are highly correlated, especially in restained tissue cores. Regional analysis also confirmed high concordance in cell density and cell composition despite discrepancies in automatic cell segmentation results within different modalities. Moreover, the multimodality alignment enabled training a deep learning model which generates high fidelity virtual H&E, providing clinical reference for MxIF images.

## Methods

### 1. Dataset and materials

#### Slide preparation, image acquisition and preprocessing.

The dataset used for this study consists of three ovarian tissue micro array (TMA) images from two tissue sections (Supplementary Fig. 1). To create this TMA, ovarian tissues were retrieved from the Mayo Clinic Ovarian Tissue Archive, punched into arrays and made into formalin-fixed paraffin-embedded (FFPE) blocks. Two continuous 5μm sections were cut for MxIF and H&E staining.

For the first section (Section 1 in Supplementary Fig. 1), a sophisticated approach involving cyclic MxIF (Multiplexed Immunofluorescence) staining was employed to meticulously assess protein expression levels within individual cells. This method enabled iterative quantification, wherein tissues were sequentially subjected to fluorescent antibody staining, digitally scanned, and then the dye was inactivated to facilitate successive rounds of staining and imaging. The raw MxIF images from CellDive were processed using our previous preprocessing pipeline to remove autofluorescence (AF) and convert into OME TIFF files. Following the completion of MxIF imaging, the restained section was restained with H&E (Hematoxylin and Eosin), providing additional morphological context to complement the molecular insights garnered through MxIF. The second section (Section 2 in Supplementary Fig. 1) only underwent H&E staining. Specifically, the MxIF images were obtained from CellDive, and H&E images were from Zeiss Axio Z1. Images from both sections were used in our method development and evaluations.

Since the TMA images consist of many FOVs (fields of view) and each FOV is a tissue core image, the TMA images were de-arrayed within QuPath [12] for downstream alignment and evaluation. An ID was assigned to each tissue core. The extracted TMA core images were visually inspected and the tissue cores without sufficient tissues were discarded. StarDist was used as our baseline for cell segmentation. The segmentation results were imported into QuPath for quantification. To investigate how the cell segmentation results affect the alignment, we also included a watershed threshold segmentation method for comparison.

### 2. Alignment Algorithms

#### A. Problem Formulation

Considering the scenarios in histopathology image scanning, three transformations were included in for alignment, in which scaling results from the differences of pixel spacing within two images, rotation and translation result from the differences in the tissue locations. The overall transformation from one image to another can be summarized into a transformation matrix M.


1 $$M=\left[\begin{array}{ccc} S\;*\;cos(\theta ) & -sin\;(\theta ) & dx\\ sin\;(\theta ) & S\;*\;cos\;(\theta ) & dy\\ 0 & 0 & 1 \end{array}\right]$$

In this formular, the scaling was denoted by S, rotation was denoted by θ. The translation/shifting distances on x and y directions were denoted by dx and dy, respectively. The distance of translation T can be calculated as $\sqrt{dx^{2}+dy^{2}}$.

#### B. Coherent Point Drift (CPD)

CPD was employed to generate a raw alignment for cell centroids (Fig. 2A). This method formulates the point-set registration as a probability density estimation problem, where one point cloud is represented using a Gaussian Mixture Model (GMM). Considering the transformation defined in formula (1), the original CPD algorithm needs to update three major parameters (θ,S *and* T) for the alignment. As an Estimation-Maximization (EM) method, CPD iteratively estimates the point correspondence and updates the three parameters until convergence [10].

Specifically, to reduce the degrees of freedom within the CPD algorithm, the cell centroid coordinates were converted into micrometers (μm), so the scale value within formula (1) can be set to a constant value (S=1). In our case, the pixel size of MxIF and H&E image are 0.325μm and 0.212μm, respectively. Pixel coordinates can be converted to micron units by multiplying by the pixel size.

#### C. Graph Matching (GM)

**Figure 1:**
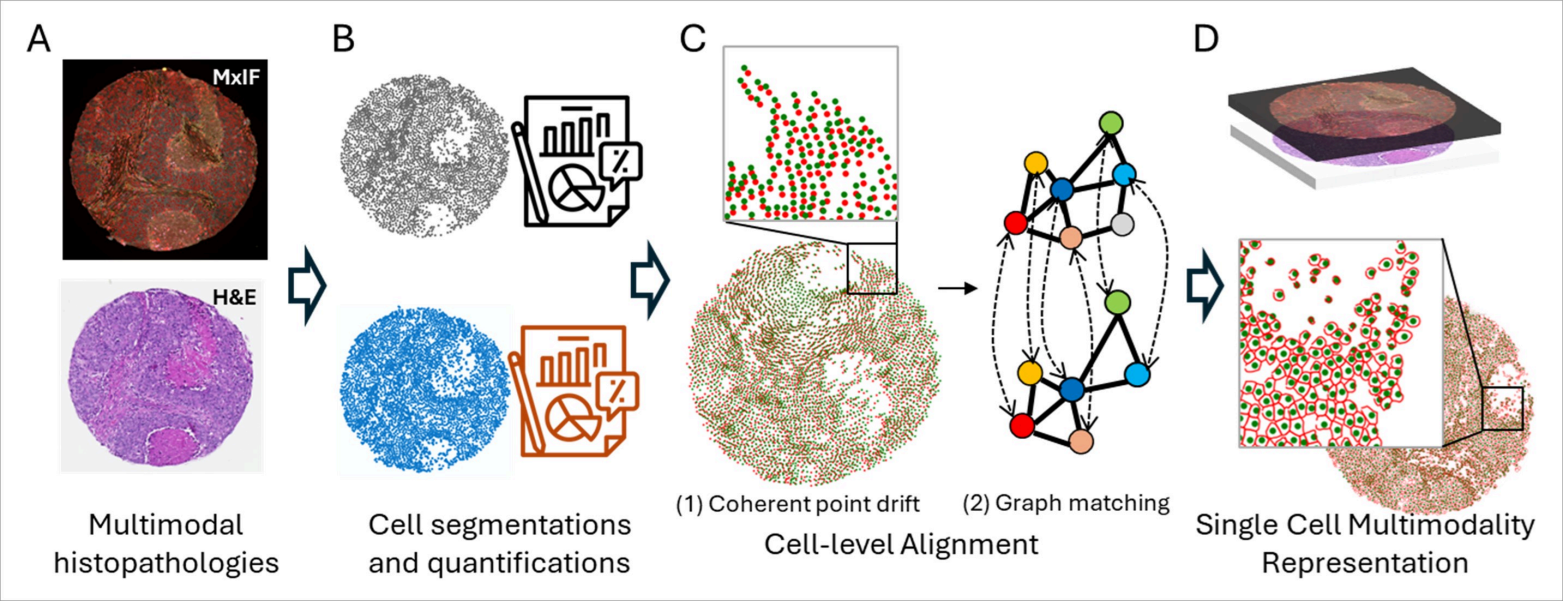
Overview of our framework. A) MxIF and H&E images to be aligned; B) Cell segmentations and quantifications; C) Cell-level alignment. 1) coherent point drift. 2) graph matching; D) Aligned MxIF and H&E images with combined cell-level representations.

Since the CPD-based method estimates the point correspondence using GMM, the alignment was estimated based on the cell densities rather than one-to-one correspondence. To calibrate the alignment, we formulated the cell-to-cell correspondence as a graph matching problem, which was implemented as a module concatenated to the CPD in our framework (Fig. 1C). In this phase, the cells from H&E or MxIF were abstracted as nodes in a mathematical graph.

##### C1. Creating subgraphs

**Figure 2:**
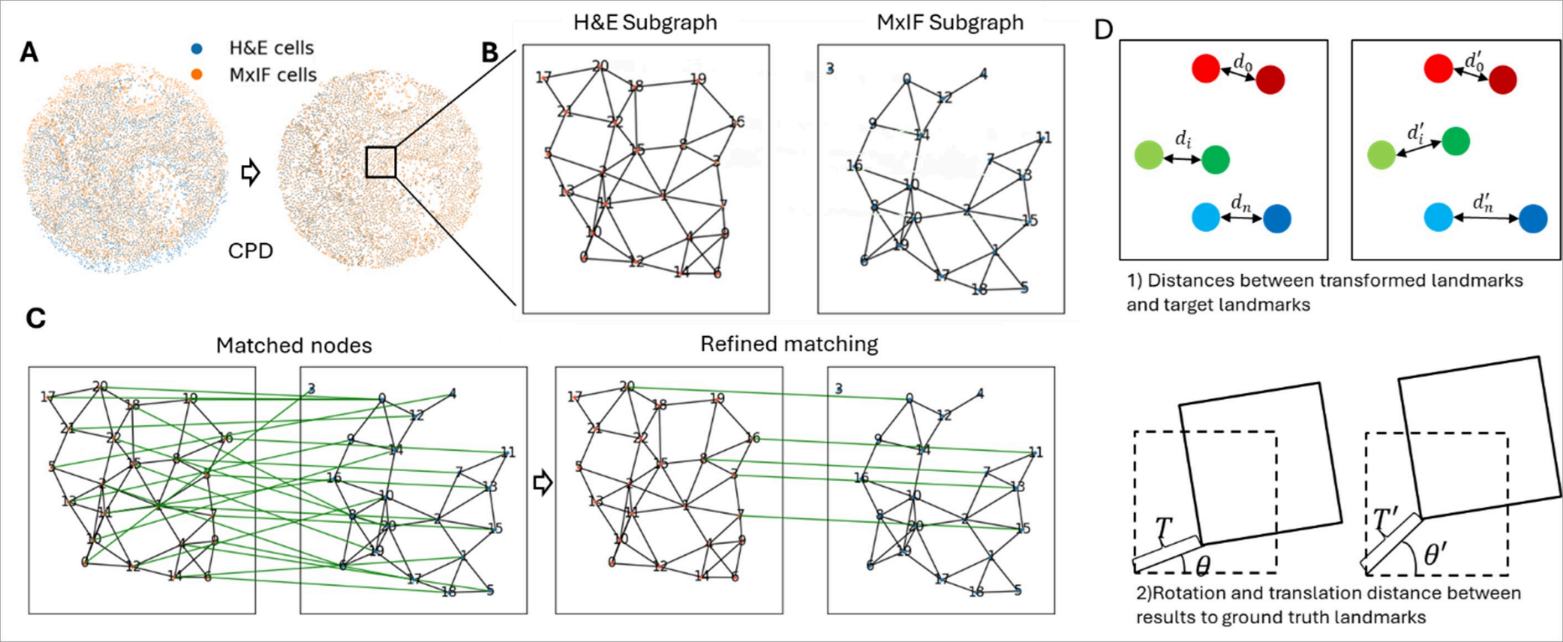
Illustration of alignment algorithms and alignment accuracy evaluation method. (A) Cell centroids were first aligned with CPD, and then fine-tuned with GM. Within the GM phase, (B) subgraphs were created for H&E and MxIF subregions with raw aligned cells as graph nodes. (C) The putative matching nodes were then filtered to establish the correct cell correspondence. (D) Alignment was evaluated with 1) Distances between landmarks after transformation (red dots) and target landmarks (green dots). 2) Rotation and translation. Landmark distances, translations and rotations were denoted by d_i_,T and θ in ground truth and d_i_’,θ’ and T’ within our results.

After the CPD transformation, the cells from H&E and MxIF were roughly aligned. Two subgraphs, the source graph from H&E and the target graph from MxIF were created for matching. Since the subregions with denser cell counts usually present magnificent tissue architectures, while the regions with sparse cells can more likely be affected by cell segmentation noise [13], the cells for graph creation were randomly sampled from subregions with dense cell distributions. Kernel density estimation (KDE) was used to calculate density value d for each cell; cells with d > = 0.5 were optional cells for sampling. To create a source graph for H&E, the cells were sampled from a window with size of 50 μm (Fig. 2B). For the target graph in MxIF, the location of the sampling window was calculated by mapping the centroid of sampling window in H&E using CPD transformation, and the window size was set to 150 μm. The edges for both source and target graphs were added based on proximity of cells if the pairwise distance was less than 15 μm.

##### C2. Node matching

Graph matching is a computational technique that seeks to establish node-to-node correspondence between two or more graphs. This process involves solving a combinatorial optimization problem, which is typically NP-hard. The goal is to match nodes from one graph to another while considering the similarity of the nodes (node affinity) and the relationships between connected nodes (edge affinity). By considering both types of affinities, graph matching methods tend to be more resilient to noise and outliers in the data, making them suitable for complex real-world applications.

In our case, the affinity matrices were built by calculating node and edge similarities between corresponding graphs. Node features, such as cell morphologies (perimeter, solidity), were used to quantify node affinity. Edge affinity was based on the distances between connected nodes, with edge features capturing the structural relationship between node pairs in the graph. A Reweighted Random Walks Matching (RRWM) solver was then used to iteratively refine the matching score based on the affinity matrix, which yields a probability distribution for potential matches. These soft matching results were converted into a final “hard” matching using a combinatorial optimization method like the Hungarian algorithm or the Sinkhorn algorithm[11, 14].

##### C3. Filtering matched node pairs

Although graph matching establishes the one-to-one correspondence between nodes in source and target graphs, not all the node correspondences are true positive. Considering the cells segmented out of the original H&E and MxIF images also represented the microstructures of ovarian tissue images, we introduced Locality Preserving Matching (LPM)[15] to exclude unreliable matching node pairs for the final spatial correspondence, as this method was explicitly designed to filter putative matching point pairs while preserving the spatial neighborhood relationship among points. Figure 2C illustrates the graph matching results before and after LPM filtering, only the matching pairs preserving the neighborhood relationships were kept. In our case, the graph nodes in the source plot were the cell centroids from H&E segmentation, while the nodes in the target plot were cell centroids from MxIF. After the matching point pairs filtering, the left point pairs were used to calculate the affine transformation that translocates H&E cell locations to MxIF cell locations. To implement our framework, three major python packages StarDist [16], probreg [17] and pygmtools [11] were used. More details about the implementations can be found in our shared GitHub code (https://github.com/dimi-lab/MultimodalityHistoComb).

### 3. Alignment Accuracy Evaluation

#### A. Creating alignment ground truth

The ground truth of cell level correspondence was established by annotating landmarks within H&E and MxIF image pairs from 20 TMA cores without significant tissue damage. For each selected pair of tissue cores, MxIF and H&E images were imported into QuPath, in which the point annotation tool was used to annotate eight pairs of landmarks (Supplementary Fig. 2). To efficiently locate corresponding cell pairs within H&E and MxIF, significant microarchitectures, such as interface of tumor and stroma, were visually referenced to navigate to individual cells. Landmark points were placed at the center of the cells close to significant microarchitectures in both H&E and MxIF. To minimize annotation errors, lymphocytes were preferred as landmarks, since their nuclei are dark and small. Then the coordinates of annotated landmarks were exported from QuPath to calculate the ground truth transformation matrix M, in which the rotation θ, scale S and translation dx and dy for the rigid alignment were summarized in formula (1).

#### B. Evaluation metrics

To quantitatively evaluate the performance of alignment accuracy, three metrics were used by referring to existing work [2], as summarized in formula (2). We compared the differences between alignment results from annotation and our methods. Following the completion of alignment, the location of landmarks annotated in the source image were mapped to the new locations within the target image, labeled with red dots in Fig. 2D. The average distance between landmarks in target image (green dots) and transformed landmarks from source image (red dots) was used to measure the overall alignment accuracy, denoted by ΔD in (2). With the annotated landmarks, the ground truth rotation angle θ and translation distance T can be calculated. The difference between the results from our method and the ground truth were the other two quantitative metrics, denoted by Δθ and ΔT respectively. Using the defined evaluation metrics, alignment accuracies were evaluated for re-stained and serial sections.


2 $$\Delta \;D=\frac{1}{n}\sum_{i=0}^{n}\left|d_{i}-d_{i}^{\prime }\right|\;\;\Delta \;T=\left|T^{\prime }-T\right|$$

$$\Delta \theta =\left|\theta^{\prime }-\theta \right|$$

### 4. Downstream Analysis

To interpret dark field MxIF images effectively, H&E-stained counterparts are commonly used to provide cell-level referencing. Ideally, the same tissue section used for MxIF should be restained with H&E after the MxIF scanning to ensure identical referencing. However, the tissue may become damaged after multiple rounds of MxIF imaging, resulting in incomplete or compromised H&E staining. Consequently, many MxIF platforms offer virtual H&E images, though the quality is often insufficient for clinical purposes. Another common approach is to use serial sections from the same tissue block for H&E staining. However, these serial sections provide a less reliable reference compared to restained H&E, as the cells do not always correspond one-to-one with those in the original MxIF images. This raises two important questions: 1) How reliable are serial section H&E images as references for MxIF analysis when restained H&E is not available? 2) Can high-quality virtual H&E images be generated from MxIF data to serve as a reliable reference, similar to restained H&E?

Using our specific ovarian TMA dataset, we demonstrated that our alignment method addresses these challenges by 1) validating cell features across MxIF and H&E (both restained and serial), and 2) facilitating the training of a deep learning model that generates high-fidelity virtual H&E images directly from MxIF image channels. This approach provides a practical solution for reliable referencing in MxIF analysis without the need for additional tissue sectioning and staining.

#### A. Cell feature concordance

Pathologists rely on tissue staining and cell morphology to make accurate diagnoses, so automating the extraction of cell features is crucial for replicating their interpretative processes, especially in large-scale studies. By analyzing the colocalization of cell nucleus staining and morphological characteristics, we can evaluate the consistency of features at both the cellular and regional levels. This ensures the quality of MxIF data and the reliability of downstream analyses.

To achieve this, cells were identified using segmentation models [7], and quantitative cellular features were extracted for both MxIF and H&E images using QuPath[18]. These features included measurements such as average DAPI or Hematoxylin signal intensity per cell and the area of the cell nucleus. Comparisons of these measurements were made either at the individual cell level or across regions for both restained and serial tissue sections. This process is critical for ensuring that cell-based analyses, including those related to tissue architecture and pathology, are robust and interpretable. Additionally, such automated feature extraction supports the scalability of pathology workflows by providing standardized, objective measurements for large cohort studies.

#### B. Generating virtual H&E from MxIF

Using our alignment method and dataset, we demonstrated the feasibility of generating high-fidelity virtual H&E images by training a deep generative model on selected image channels from existing MxIF data. The architecture of the deep learning model was presented in our previous work[19]. Since DAPI (4’,6-diamidino-2-phenylindole), NAK (Na/K-ATPase) and PANCK (pan-cytokeratin) are three important markers for nucleus and membrane staining, these three channels were extracted from MxIF as one of the inputs (condition) for training the conditional Generative Adversarial Network (cGAN). In the training phase, the generated image (virtual H&E) from the generator of cGAN was compared with the real H&E (Fig. 5A). In the testing phase, the qualities of generated virtual H&E images were both quantitatively evaluated using quality metrics and visually inspected by pathologists. Using HoverNet [20] as the automatic cell classification model, downstream cell composition was also analyzed to evaluate the consistency of cell population between the real and virtual H&E images.

## Results

### 1. Our framework achieves cell scale alignment for MxIF and H&E images.

**Figure 3:**
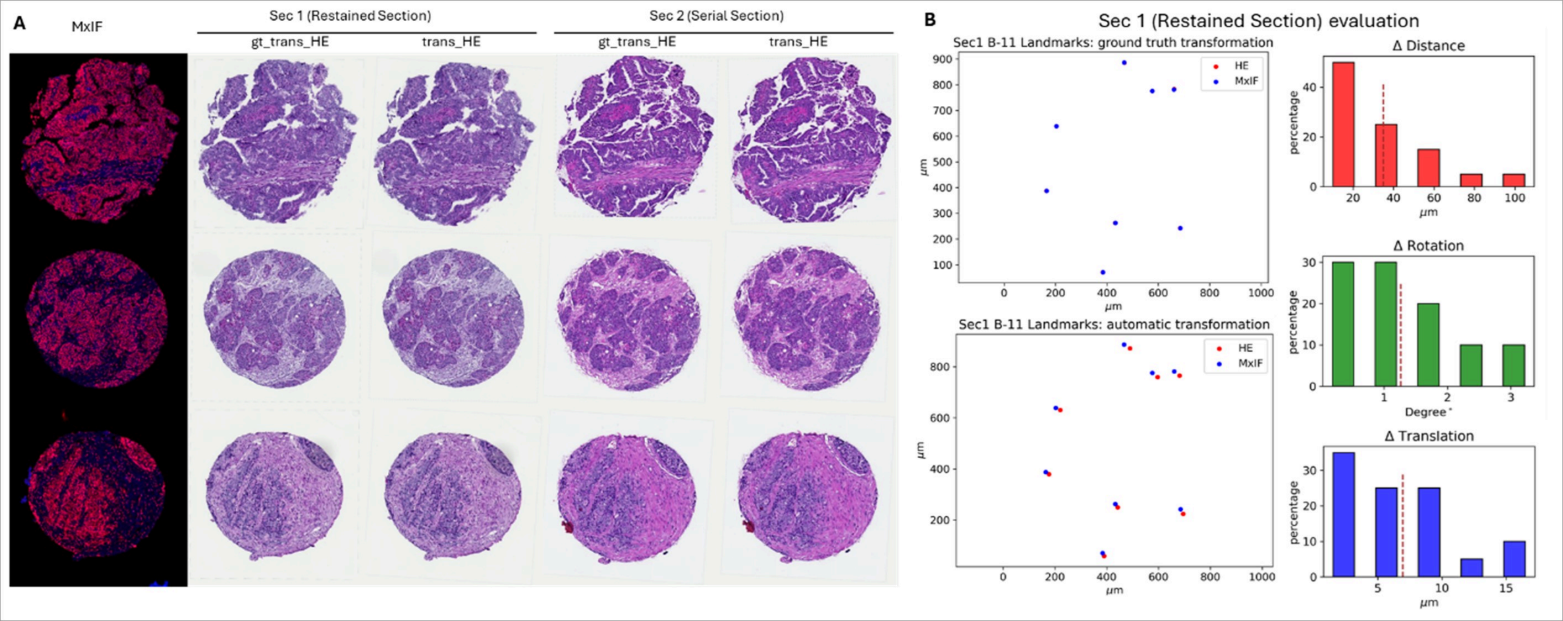
Qualitative and quantitative evaluation results. A) Examples of MxIF and aligned H&E for both restained and serial tissue sections. B) A restained section example of aligned landmarks obtained from annotation (ground truth) and our (automatic) method. The right side is the quantitative evaluation metrics in the all the annotated restained cases.

Our method was tested on both restained and serial sections. For each section and each core, the H&E images were aligned to MxIF. According to the landmark annotations, ground truth transformation M was calculated and applied to the restained and serial section. The ground truth results H&E are visualized in Fig. 3A column 2 and 4, while the results from our method are visualized in column 3 and 5. For both sections, our H&E results were visually aligned well to the corresponding ground truth H&E, even for the broken-up tissue core, shown in the first row of Fig. 3A. Meanwhile, our H&E results for both sections aligned well to the MxIF, even though there were micro architecture and staining color differences between the two sections.

To visually check the alignment accuracy for the restained sections, the landmarks from H&E (red dots) were translocated according to manually annotation and our automatic method respectively, and then plotted together with landmarks from MxIF (blue dots). As an example (tissue core ID: B-11) shown in Fig. 3B, the MxIF landmarks were fully overlayed on translocated H&E landmarks, indicating that for the restained section and manual annotation, the alignment error was minor. The scatter plot also indicated that the automatic alignment errors were higher than the ground truth alignment. According to the definition of our evaluation (formular (2)), the histograms of the quantitative metrics were plotted in Fig. 3B, with $\Delta {\;D}_{\sec1}\;=\;32.47\;\pm \;27.25$, ΔT=6.94±4.73 and Δθ=1.26±0.98. Average values were labeled withred dash line.

**Figure 4:**
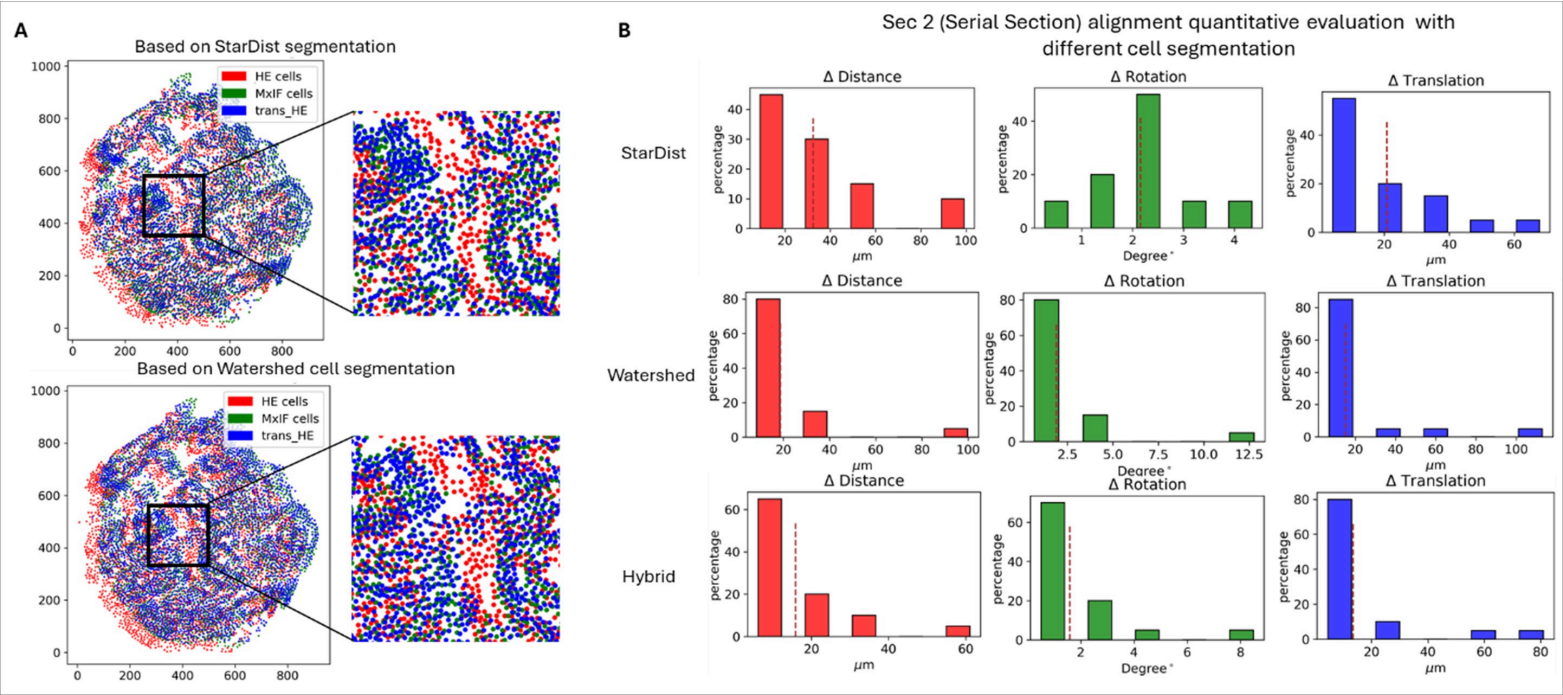
Results of alignment initiated from different cell segmentation methods. A) Cell centroids scatter plot for MxIF (green), original H&E (red) and transformed H&E (blue) from a serial section example. B) Distribution of quantitative metrics for evaluating alignment accuracy using different cell segmentation results as the starting point. First row: StarDist; Second row: Watershed; Third row: StarDist for MxIF while Watershed for H&E.

With the same settings, the alignment accuracy was also evaluated on the serial sections. Results were shown in the first row of Fig. 4B, with $\Delta {\;D}_{\sec2}\;=\;34.47\;\pm \;25.71$, T=20.77±17.46 and Δθ=2.15±0.97, suggesting that both restained and serial sections can be aligned well with manual annotation, but alignment errors were higher for serial if measured with rotation and translation.

### 2. Our framework is portable to different segmentation models.

Since our method relies on cell segmentation, we investigated the alignment performance with respect to two different segmentation methods, StarDist and Watershed. As the restained tissue section provides more reliable one-to-one cell correspondence, our evaluations were conducted on Section 1. Although Watershed generates more over- and under- segmentation cell instances [21], a similar transformed H&E image can be obtained by applying the transformation from our method. Taking the same tissue core (ID: B-11) as an example, the discrepancies between aligned H&E results images can only be observed by zooming into the cell level and visualizing the cell centroids before and after transformation (Fig. 4A). Moreover, according to the quantitative evaluation metric ΔD, the performance of using Watershed was even better than that using StarDist (Fig. 4B).

We also tested the method using hybrid results from two cell segmentation models, with StarDist for MxIF and Watershed for H&E. Compared to the default segmentation method StarDist, evaluation metric ΔD was lower when using hybrid cell segmentation results for alignment with Fig. 3B, with $\Delta {\;D}_{\mathrm{StarDist}}\;=\;32.47\;\pm \;27.25\;vs$. $\Delta {\;D}_{\mathrm{hybrid}}\;=\;15.87219264\;\pm \;14.65349468$. The performance fluctuations shown in Fig. 3B and Fig. 4B may result from the inconsistencies within H&E and MxIF cell segmentation. Since there were many cell segmentation models available specifically for H&E or MxIF, our method provided a straightforward and portable way to align cells using segmentation results as the starting point for alignment.

### 3. Multimodality alignments enable cell and regional feature concordance evaluation.

**Figure 5:**
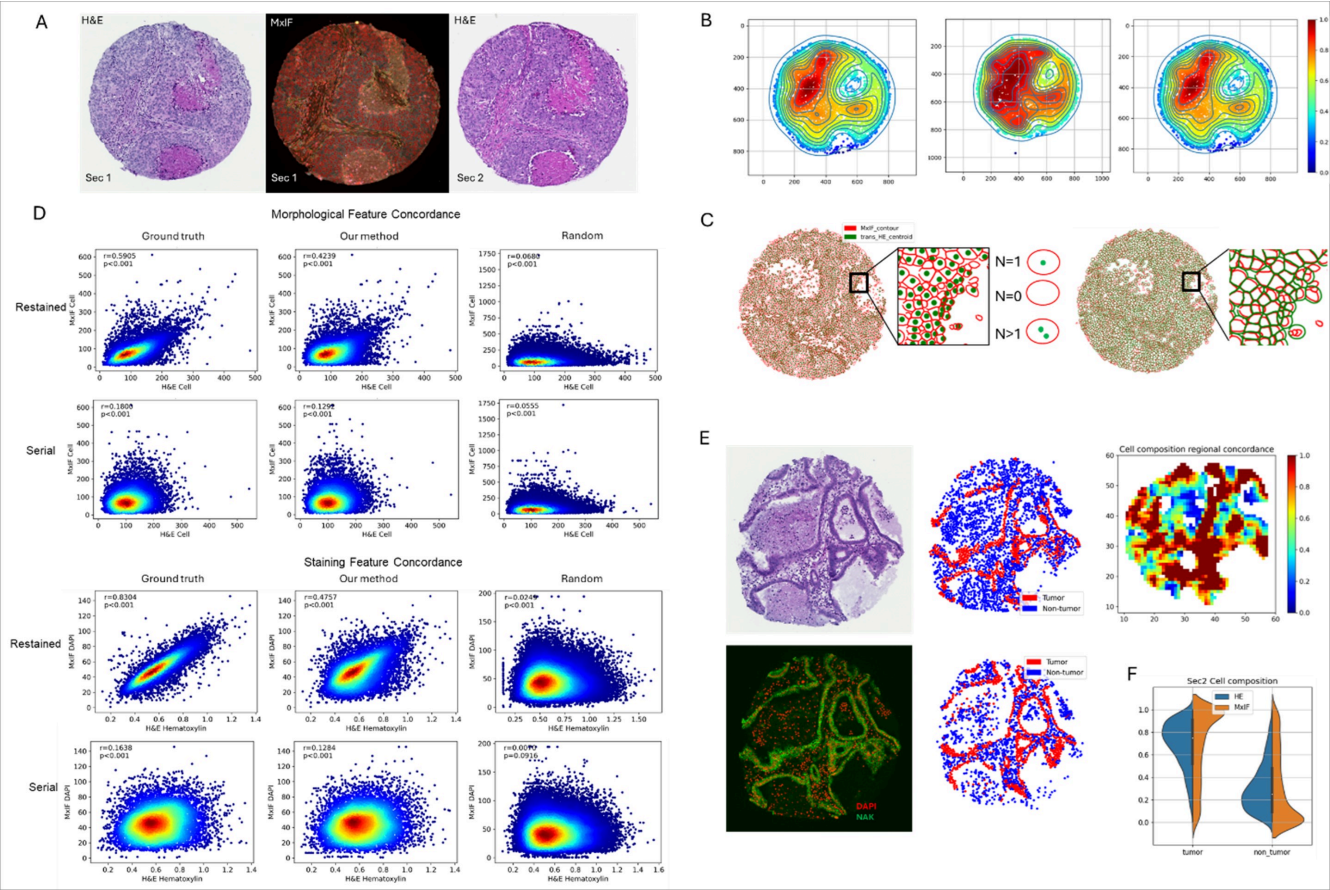
Visualization of aligned cells, cell feature concordance evaluations and cellular composition comparison. A) An example of aligned MxIF and H&E, including both a restained and a serial section. B) Regional cell density for the example. C) An example of aligned cells with cells from MxIF (red) showing cell contours while the cells from H&E(green) show cell centroids (left of the figure). The same example with cells from both H&E (green) and MxIF(red) showing cell contours (right of the figure). D) Cell feature concordance under different conditions. Row 1 and 3: restained section; Row 2 and 4: serial sections; Column 1: aligned with manual annotation; Column 2: aligned with our method; Column 3: randomly align to a tissue core. E) An example of H&E and MxIF. The cells were labeled with red (tumor) and blue (non-tumor). Regional cell composition similarity was illustrated with heatmap (warm color denotes high concordance). F) The comparison of cell composition within H&E and MxIF for all serial tissue cores.

Since our alignment framework aims to enable referenced spatial analysis within the aligned tissue space, cell and regional signal concordances after the alignment were evaluated. By applying the transformation to the cell segmentation results, cells from H&E and MxIF can be aligned to the same space, as shown in Fig. 5A. Although the regional cell densities for MxIF can be higher than that in H&E due to higher cell detection sensitivity, cell densities for H&Es from different sections were almost identical, as shown in Fig. 5B. This fact implies that even if we apply the ground truth transformation, rigid one-to-one cell correspondence cannot always be established for two major reasons: 1) The cells within tissue section images were not identical, especially for serial sections. Ideally, for the restained tissue, the cells should be the same for different imaging techniques. However, there could be cells washed off or even large pieces of tissue damaged during the stain-restain process. 2) The cell segmentation models do not always generate the same segmentation results from different modalities as the models were trained from and applied to different modalities independently. For example, there are pretrained StarDist models available for H&E and MxIF respectively, but the models were trained from different datasets. The results will be different when the model is applied to H&E and MxIF independently.

To check the single-cell-level feature concordance between H&E and MxIF, the restained tissue section was used. By visualizing the aligned cells into the same space, as shown in Fig. 5C, cells from two modalities demonstrated three scenarios in single cell level correspondences: 1) N = 1, the cells are one-to-one corresponded; 2) N = 0, a cell detected in one image did not correspond to any cell in another image; 3) N > 1, more than one cell in one image corresponded to one cell in another. According to our observations, for both N = 0 and N > 1, the mismatch was caused by segmentation inconsistency between H&E and MxIF. The StarDist model we applied to MxIF was sensitive to DAPI signals, which led to two consequences: 1) For cells with weak DAPI signals, the instances detected in MxIF could be recognized as fragments within H&E which can be filtered out within segmentation process (N = 0). 2) For cells close to each other and with strong DAPI signals, multiple instances that are recognizable in H&E can be detected as single cell in MxIF (N > 1) (supplementary Fig. 3). Since N = 0 and N > 1 were extreme cases for cell segmentation, we investigated the morphological and staining feature concordance with one-to-one cell correspondence (N = 1) within all 20 evaluation cores. As shown in Fig. 5D first row, morphological features, such as cell area, from H&E cells and MxIF cells were highly correlated. Meanwhile, the staining concordances were compared on DAPI and hematoxylin as both are cell nucleus pigments (Fig. 5D third row). For both morphological and staining features, r and p values for Pearson correlation were calculated and shown in the plots.

To be more generic, we investigated the cell level feature concordance under two different conditions: 1) align with manually labeled landmarks vs. our automatic alignment; 2) serial vs. restained section. Each tissue core was also aligned to a randomly chosen core as the baseline for comparison. Specifically, after alignment, each cell in H&E was corresponded to the nearest MxIF cell for feature comparison. As shown in Fig. 5D second and forth row, both morphological (cell area) and staining features (DAPI vs. Hematoxylin) demonstrated higher feature concordance than the serial section. Though the concordance based on our alignment was lower than the ground truth, it is comparable in both restained and serial section and was significantly higher than random alignment.

Although the cell-level feature concordance in serial sections was lower, similar tissue structures in H&E and MxIF were observed. As shown in Fig. 5E, cells within H&E and MxIF were classified as tumor or non-tumor by referencing the cell features extracted by StarDist models and QuPath[21, 22]. Then, the concordance of cellular composition was evaluated by comparing proportions of tumor cells region-by-region. A heatmap was used to demonstrate the reginal similarity of cell composition, in which warm color indicates high concordance. The most discordant regions significantly overlapped with areas where H&E cell segmentations differed from MxIF cell segmentations. Cell composition differences were also compared for all serial tissue cores. The results indicated that the cell population share similar distribution but more cells were detected within MxIF images (Fig. 5F).

### 4. Alignment enhanced clinical referencing through generating high quality virtual H&E

**Figure 6:**
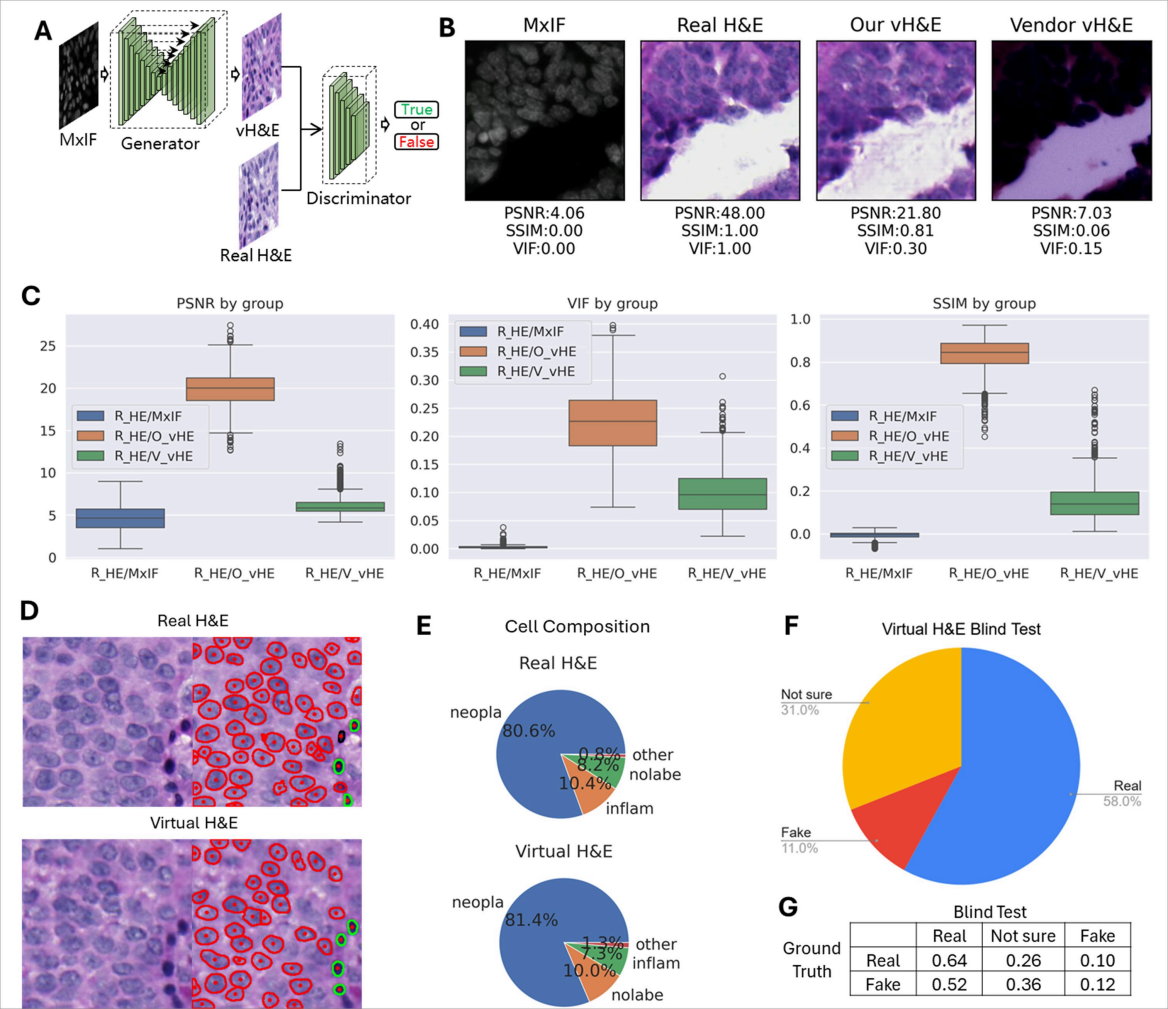
Generating virtual H&E from MxIF and image quality evaluation. A) The schema of the virtual H&E generation model. B) Example image of original MxIF, restained H&E, virtual H&E from our method and virtual H&E from the platform vendor. C) Quantitative evaluation results showing PSNR, VIF and SSIM by different groups. R_HE/MxIF denotes real H&E vs. MxIF, R_HE/O_vHE denotes real H&E vs. our virtual H&E, R_HE/V_vHE denotes real H&E vs. vender’s virtual H&E. D) Example of original real H&E and our virtual H&E images, together with cell detection and classification results overlay. E) Cell composition comparison between real H&E and our virtual H&E on our entire dataset, in which nolabe denotes no label, inflam denotes inflammatory, neopla denotes neoplastic. F) The proportion of “real”, “not sure” and “fake” images in the blind test. G) Comparison between blind test and ground truth.

With aligned real H&E and MxIF image pairs as input (Fig. 6A), the trained cGAN model can generate high quality virtual H&E images from DAPI, NAK and PANCK channels within MxIF[19]. The model was trained on 20 aligned TMA cores (5845 patches) and evaluated on a hold-out set of 10 cores (2683 patches). Since the training dataset is small, the model was trained on one GPU server and converged in 28 epochs. According to a pathologist’s visual inspection, the virtual H&E images from our model were significantly better than the vendor’s virtual H&E which was dark and did not allow visualization of cell details (Fig. 6B). Meanwhile, our virtual H&E preserved the tissue context well but generated some light tissue fragments in blank areas which might be the result of the model picking up weak background fluorescence signal from MxIF images. Three evaluation metrics, including PSNR (Peak Signal-to-Noise Ratio) [23], VIF (Visual Information Fidelity) [24] and SSIM (Structural Similarity Index Measure)[25] were also used to intrinsically measure the quality of virtual H&E images by comparing them to the corresponding real H&E[19]. Our boxplots indicated that our virtual H&E achieved the highest similarity to the real H&E (Fig. 6C).

To extrinsically evaluate the quality of generated H&E images, cell compositions within real and our virtual H&E were compared. A pretrained model MoNuSAC [26] derived from HoverNet [20] was used to classify cells in both real and virtual H&E (Fig. 6D). Using the default configuration, the detected cells were classified into four groups: inflammatory, neoplastic, other, and no label. Based on the automatic cell classification, we observed that real H&E and virtual H&E share a similar cell population, with 3.1% difference in inflammatory cells, and less than 1% difference in neoplastic (Fig. 6E). The similarity of cell composition between real H&E and virtual H&E suggested that the quality of the image generated by our model is high for providing clinical references.

A blind evaluation was designed to validate the fidelity of the virtual H&E from our method. 200 images (50% real H&E and 50% our virtual H&E) were randomly selected from our dataset and loaded into a Google Form. Without knowing the proportion of real images, pathologists were invited to label each image to “real”, “not sure” or “fake” according to their impression to the image. The summarization of this blind test indicates that 58% of images were considered to be “real”, though only 50% are from real H&E (Fig. 6F). Further investigation found that 52% of our virtual H&E passed the test. Based on the pathologist manual review, many virtual H&Es were essentially indistinguishable between the real and fake especially for the ones with considerable cellular compositions as well as acceptable resolutions. For both real and virtual H&E, many of uncertainties arose from images that were blur/out of focus, as well as those with low cellularity, increased stromal content, or other non-cellular components.

## Discussions

We introduced a novel histopathology image registration framework that relies solely on cell segmentation results, which are prerequisites for many downstream analyses. This approach allows for the integration of cell features from multiple histopathology modalities without requiring significant modifications to existing analysis pipelines. Our method is robust, as the convergence of the CPD (Coherent Point Drift) algorithm primarily depends on regional cell densities. Furthermore, we demonstrated the portability of our approach across different segmentation techniques. Although the graph matching step operates under strict conditions, it opens the door to incorporating advanced techniques, such as graph neural networks, to measure the similarity between nodes and establish cell correspondence. Developed and evaluated on a specific dataset, our work not only showed promising alignment accuracy but also highlighted potential pitfalls that can arise under various tissue cutting and staining conditions.

While existing approaches often treat histopathology image alignment as a non-rigid registration problem due to tissue warping [27], we simplified TMA (tissue microarray) image alignment as a rigid transformation. There are two main reasons for this: first, compared to whole-slide images, TMA core images are much smaller (~ 40,000 pixels in height vs. ~4000 pixels in height). Based on our observations, significant tissue warping is more prevalent in large tissue sections than in TMA cores. Second, tissue warping should ideally be avoided during slide preparation to ensure reliable downstream analysis, as non-rigid registration can only adjust for the warping rather than eliminate alignment errors. In fact, the discontinuous control points used in non-rigid registration methods can cause regional distortions [28].

Our alignment method’s reliance on cell segmentation makes it sensitive to the number of cells present, which poses a challenge when applying the technique to whole-slide images that may contain hundreds of times more cells than a TMA core (Supplementary Fig. 4A). Although CPD can be accelerated using specific optimization techniques[9], applying it to whole-slide images remains computationally demanding. To address this, we extended our method with the “Super-cell” concept [29] by clustering cells based on proximity before applying our registration approach (Supplementary Fig. 4B, C). Preliminary evaluations showed that the alignment achieved accuracy comparable to that seen with TMA cores (Supplementary Fig. 4D), indicating the potential applicability of our method to whole-slide images. Nevertheless, histopathology image alignment is particularly meaningful for TMA slides, as TMA scanning is a more practical imaging method for large cohort studies[30]. Annotating TMA core images requires only two landmarks per image pair to estimate the transformation in Eq. (1), resulting in a lower annotation workload compared to whole-slide images. Additionally, whole-slide images are more prone to regional distortion and tissue folding, making seamless image stitching more challenging even after local alignment calibration.

By aligning both restained and serial H&E section to MxIF, our work provided significant insights to H&E referenced MxIF interpretation. At the cellular level, cell morphology features were found to be more reliable in H&E images based on pathologists’ observations. In our dataset, some cells identified in MxIF appeared as fragments in the H&E images, while others that seemed to be a single cell in MxIF were actually two or three distinct cells in H&E, likely due to strong fluorescence signals spilling over into adjacent cells. Furthermore, our evaluation of cell feature concordance revealed that the agreement between restained H&E and MxIF was much higher than between serial H&E and MxIF, suggesting that restained H&E is preferable for cell-level referencing in MxIF analysis. At the regional level, we observed similar cell densities between restained and serial H&E, and both showed comparable regional cell composition with MxIF, albeit with some overall differences. Discrepancies in cell segmentation and tissue classification introduced uncertainty in combined downstream analysis, but also provided an opportunity to cross-validate image quality once the images were aligned.

Our evaluations at both the cellular and regional levels suggested that restained H&E offers a more reliable reference for MxIF analysis. Consequently, we explored the possibility of generating high-quality virtual H&E images from MxIF data. This approach not only eliminates the need for additional tissue sectioning, staining, and scanning but also preserves cell-level similarities, making it suitable for referenced MxIF study. Although training a GAN-based model is notoriously challenging, we encountered relatively few difficulties during the training phase, indicating that our method could potentially be generalized to other tissue types, provided suitable datasets are available for fine-tuning.

Upon closely inspecting the model outputs, some defects were observed: 1) The model occasionally generated artifacts in tissue regions, which is a common behavior in generative models and could potentially be used to identify image quality issues within certain MxIF markers. 2) While the color of the virtual H&E images closely resembled that of real H&E, the cell details were generally less sharp, which may contribute to lower accuracy in automated cell segmentation and classification compared to real H&E. Overall, while the virtual H&E images from our method were not as impeccable as the restained H&E, they provided exemplary MxIF referencing with significantly higher quality than the default virtual H&E from the platform.

Our work creates new opportunities for learning latent information from multiple modalities of histopathology images in an integrated way. Within co-localized histopathology images, we can utilize complementary information from each modality to enrich cell-level and regional representations. The success of models like CLIP [31] for text-image learning inspired us to explore the possibility of applying similar approaches for jointly learning from multiple histopathology modalities. However, the design of current text-image models may not be ideally suited for the medical domain, where clinical notes generally offer an overall summary of a patient’s condition, while histopathology image tiles capture highly localized information. This disparity in data granularity poses a challenge for existing text-image alignment methods, which are limited by these global versus local perspectives. In contrast, when dealing solely with image data, aligning different modalities resolves the granularity gap, allowing for more refined co-training approaches, such as patch-to-patch or even cell-to-cell alignment. Our method thus lays the groundwork for more precise multi-modal learning, enabling new downstream applications that can fully exploit the complementary nature of different histopathology imaging modalities.

## Conclusion

Our work provides a robust, scalable solution for aligning multimodal histopathology images at the cellular level, enabling integrative analyses that combine the strengths of H&E and MxIF modalities. By demonstrating high alignment accuracy, facilitating virtual H&E generation, and uncovering feature concordance across modalities, this framework lays the foundation for more advanced multimodal workflows in histopathology research and clinical applications.

## Data Availability

The dataset used in this study is publicly available at https://immunoatlas.org/MYCB/240802-1/MYCB24004/ Code and documentation for the proposed framework are accessible via GitHub: https://github.com/dimi-lab/MultimodalityHistoComb Details of the virtual vs. real H&E blind test can be found in this Google Form: https://docs.google.com/forms/d/e/1FAIpQLScAYf_t8eBe2Oo6ac3SWS6i9QstyUOVQqT1GEobic05gmlhKA/viewform?vc=0&c=0&w=1&flr=0
